# Hydrostatic pressure can induce apoptosis of the skin

**DOI:** 10.1038/s41598-020-74695-5

**Published:** 2020-10-19

**Authors:** Tien Minh Le, Naoki Morimoto, Nhung Thi My Ly, Toshihito Mitsui, Sharon Claudia Notodihardjo, Maria Chiara Munisso, Natsuko Kakudo, Hiroyuki Moriyama, Tetsuji Yamaoka, Kenji Kusumoto

**Affiliations:** 1 Department of Plastic and Reconstructive Surgery, Kansai Medical University, Hirakata, Osaka Japan; 2 Department of Plastic and Reconstructive Surgery, Graduate School of Medicine, Kyoto University, Kyoto, Japan; 3 Department of Dermatology, Kansai Medical University, Hirakata, Osaka Japan; 4 Pharmaceutical Research and Technology Institute, Kindai University, Higashi-osaka, Osaka Japan; 5 Department of Biomedical Engineering, National Cerebral and Cardiovascular Center Research Institute, Suita, Osaka Japan

**Keywords:** Biomaterials, Tissue engineering

## Abstract

We previously showed that high hydrostatic pressure (HHP) treatment at 200 MPa for 10 min induced complete cell death in skin and skin tumors via necrosis. We used this technique to treat a giant congenital melanocytic nevus and reused the inactivated nevus tissue as a dermis autograft. However, skin inactivated by HHP promoted inflammation in a preclinical study using a porcine model. Therefore, in the present study, we explored the pressurization conditions that induce apoptosis of the skin, as apoptotic cells are not believed to promote inflammation, so the engraftment of inactivated skin should be improved. Using a human dermal fibroblast cell line in suspension culture, we found that HHP at 50 MPa for ≥ 36 h completely induced fibroblast cell death via apoptosis based on the morphological changes in transmission electron microscopy, reactive oxygen species elevation, caspase activation and phosphatidylserine membrane translocation. Furthermore, immunohistochemistry with terminal deoxynucleotidyl transferase dUTP nick-end labeling and cleaved caspase-3 showed most cells in the skin inactivated by pressurization to be apoptotic. Consequently, in vivo grafting of apoptosis-induced inactivated skin resulted in successful engraftment and greater dermal cellular density and macrophage infiltration than our existing method. Our finding supports an alternative approach to hydrostatic pressure application.

## Introduction

High hydrostatic pressure (HHP) is a physical technique for quickly inactivating tissues or organs without using any chemical reagents and it has recently been used to prepare decellularized tissues in the biomedical engineering^1–3^. Decellularized tissue retains its native structure or mechanical properties, making it an ideal substitute or scaffold for tissue engineering and regenerative medicine. However, the decellularized tissue prepared from inactivated tissue after removing the cellular remnants is believed to cause inflammation in in vivo implantation^4^.

In our previous studies, we reported that HHP at 200 MPa for 10 min could induce cell death without damaging the extracellular matrix (ECM) of the skin^5^, and we applied this technique in a clinical trial to treat giant congenital melanocytic nevus (GCMN). In that trial, we attempted to transplant the inactivated nevus tissue by HHP without removing the cellular remnants in combination with cultured epidermal autograft (CEA)^6,7^. This is because it takes several days to remove the cellular remnants from the inactivated skin, and this washing process usually damages the matrix to some degree. In our preclinical study, the CEA was able to survive on the inactivated skin in immunodeficient mouse models. The engraftment of inactivated skin was superior to the decellularized skin using a porcine model^8–10^. However, it was reported that the success rate of CEA is insufficient on inflammatory tissue, such as granulation tissue^8,9,11^. As a result, we planned to perform CEA on the inactivated nevus which contains plenty of inactivated nevus cells, and therefore the degree of induced inflammation is a concern for such a clinical study.

We recently showed that HHP at 200 MPa for 10 min induced cell death mostly via necrosis^12^. In the necrotic pathway, the cell membrane rapidly becomes permeable, leaking the intracellular contents and immunogenic cellular material into the local environment^13^. Without appropriate washing, such material may generate a strong immune reaction following implantation and subsequently lead to rejection of the graft^14,15^. Therefore, to reduce the inflammation, we explored whether or not we could inactivate skin tissue by HHP through the apoptotic pathway.

Apoptosis or programmed cell death (PCD) is recognized by cell rounding, DNA fragmentation, externalization of phosphatidylserine (PS), and caspase activation. Apoptotic cells show a series of physical changes, such as plasma membrane blebbing, permeabilization of the mitochondrial outer membrane, nucleus disintegration and eventually cell disintegration into apoptotic bodies that are then engulfed and degraded by phagocytes^13,16–18^; inflammatory reactions of the implanted grafts are thereby decreased during the apoptotic process. Regarding the pressurization conditions for apoptosis, it has been reported that HHP of ≤ 100 MPa cannot inactivate cells^19^. However, Ogino et al. recently demonstrated that HHP at 50 MPa for 24 h could prolong the generation time and stop microbial proliferation^20^. Therefore, in this study, we focused on HHP at 50 MPa, and we pressurized human fibroblasts at 50 MPa up to 48 h and evaluated the viability and death pathways. We then explored the inactivation of skin at 50 MPa and the engraftment outcome of that inactivated skin.

## Results

### PS externalization and membrane permeability

**Figure 1:**
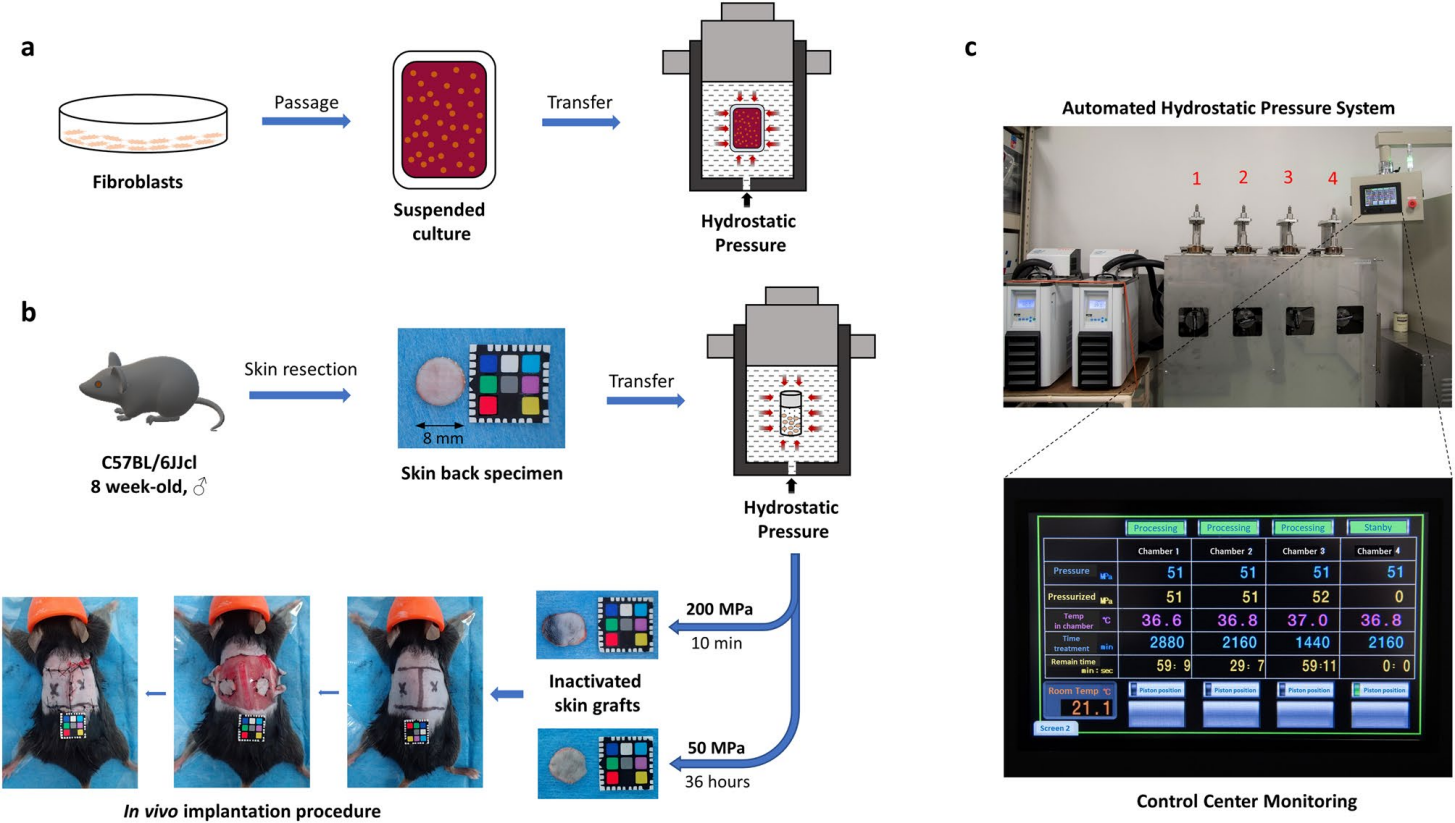
Schematic description of the pressurization process. (**a**) Preparation of suspended culture fibroblasts in a plastic bag and the induction of hydrostatic pressure inside the isostatic chamber. (**b**) Procedures for inactivating skin tissues by HHP and subcutaneous transplantation onto the backs of mice. A 1 cm × 1 cm color chart was used as a scale bar. (**c**) The new custom HHP device, comprising a temperature management system, four separate isostatic chambers for input objects (No. 1–4) and an automated touch screen control center.

To test the hypothesis that HHP at 50 MPa is capable of inducing apoptosis, a fibroblast cell line was developed in a suspended culture in sealed bags filled with DMEM/10% fetal bovine serum (FBS) and pressurized under certain conditions, as described in Fig. 1. We detected apoptotic cells using Apopxin Green (FITC) and 7-AAD staining (ab176749; Abcam).

**Figure 2:**
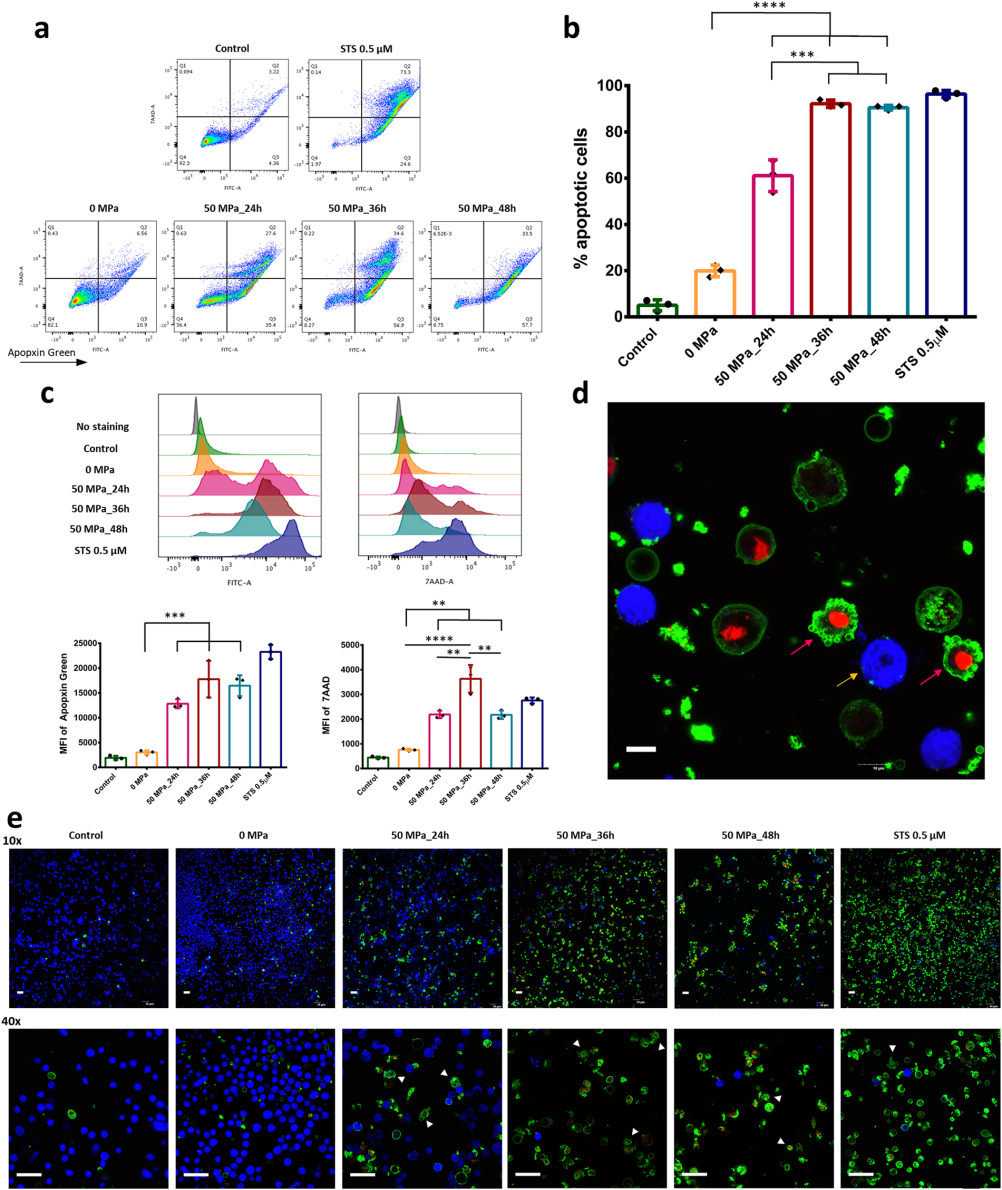
Detection of apoptotic cells by PS externalization and membrane permeability. (**a**) Fibroblast cells were treated with 50 MPa HHP for different durations or with STS. Each cell is represented by a dot plot in the flow cytometer diagram formed by the combination of Apopxin Green-FITC and 7-AAD immunofluorescence. (**b**) The comparison of total apoptotic cells among groups. The control and STS treatment groups are not compared (n = 3). (**c**) Representative histograms of different fluorescence expression values and the comparison of the mean fluorescence intensity (MFI) of Apopxin Green (FITC) and 7-AAD (n = 3). (**d**) Immunofluorescence image of apoptosis/necrosis staining of cells treated by HHP for 36 h. Magnification 100×, scale bar: 10 μm. (**e**) Immunofluorescence images of apoptosis/necrosis staining show the difference in the density of viable cells (blue color) and apoptotic cells (green/red color) or dead cells (red color) in small magnification (10×, scale bar 50 μm) among groups. At higher magnifications (40×, scale bar 20 μm), the morphological characteristics of other stages of apoptotic cells in each group can be observed, particularly the release of apoptotic bodies (white arrowhead). Data are representative of at least three independent experiments.

As shown in Fig. 2a, the early apoptosis (FITC^+^/7-AAD^−^) and late apoptosis (FITC^+^/7-AAD^+^) populations were slightly larger in the 0 MPa group than in the control group. However, under pressurization, those populations showed a further increase after 24 h, and most were recognized as apoptotic (FITC^+^) after 36- or 48-h treatment (Fig. 2a). On comparing the ratio of apoptotic cells among groups (Fig. 2b), we found visible differences between the 0 MPa and 50 MPa groups (p < 0.0001), thus proving that this pressure level effectively induced apoptosis in a fibroblast cell line. However, the most effective treatment condition differed depending on the exposure duration. After 36 or 48 h, more than 90% of cells were apoptotic, compared to the 24-h group, with around 60% apoptotic cells (p < 0.001). Figure 2c shows markedly different peak histograms and mean fluorescence intensity (MFI) values for Apopxin Green-FITC and 7-AAD among the control and 0 MPa groups and other pressure conditions.

On immunofluorescence imaging of apoptosis/necrosis staining (Fig. 2d), we visibly identified early apoptotic cells (Apopxin Green^+^/7-AAD Red^−^), late apoptotic cells (Apopxin Green^+^/7-AAD Red^+^) and even apoptotic bodies (small green objects) with living cells (Cytocalcein 450 Blue^+^, yellow arrow). Furthermore, we also detected the unique stage of the release of apoptotic bodies from late apoptotic cells (pink arrow). Figure 2e shows the total stained population of all groups at 10× magnification, in which we can differentiate the number of living cells (blue stained) and apoptotic cells (green/red stained) among the groups. At higher magnifications (40×), we are able to observe in greater detail the morphology of apoptotic cells with PS exposure, showing an intact membrane, an increased permeability and the release of apoptotic bodies in the final stage of apoptosis (white arrowhead).

### TEM observation of HHP-treated fibroblast cells

**Figure 3:**
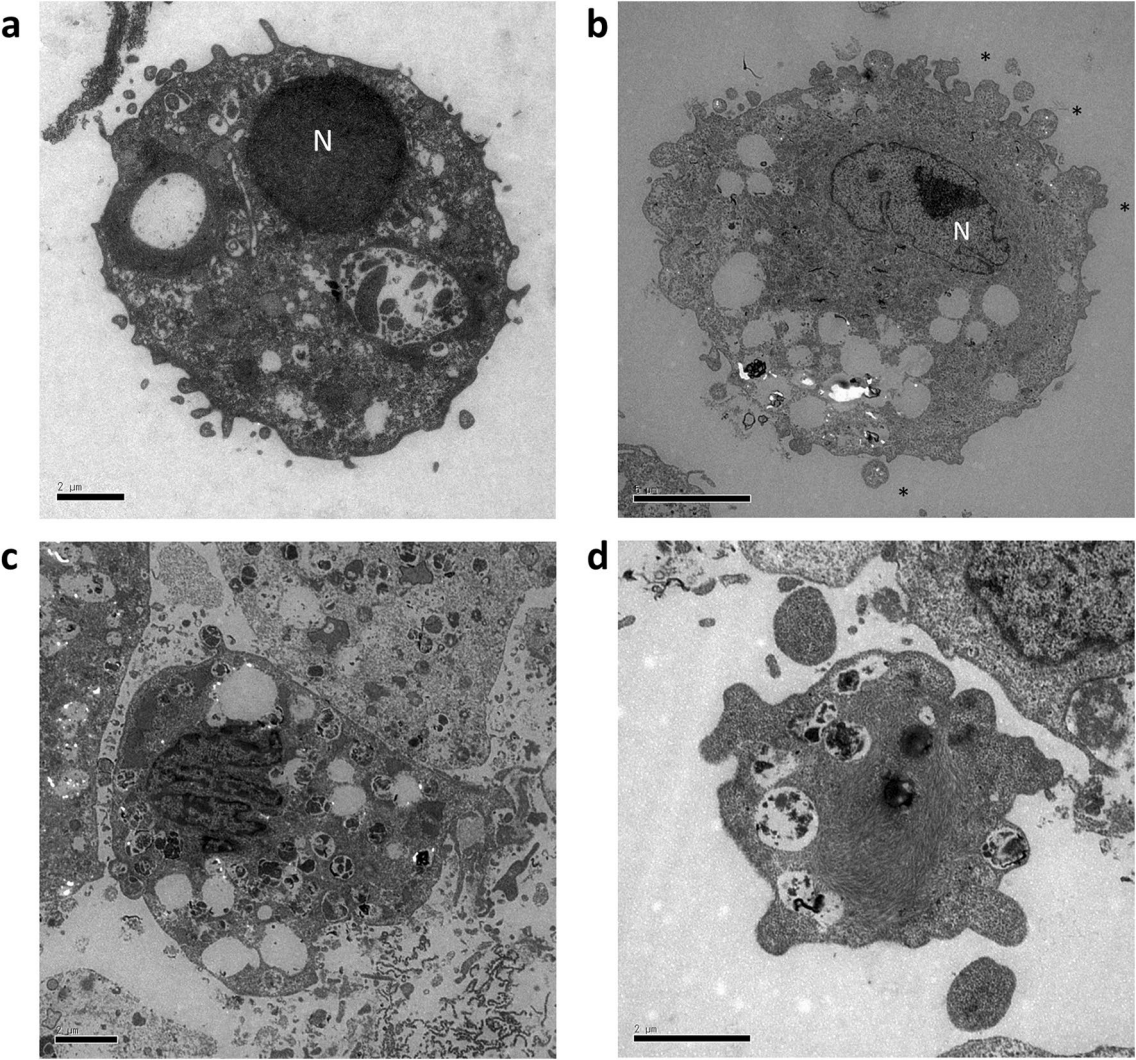
Transmission electron microscopy (TEM) appearance of HHP-treated fibroblast cells. (**a**) Untreated cells (0 MPa) exhibited a normal morphology with intact membrane, scant cytoplasm and round nuclei (N). (**b**) Cells subjected to HHP 50 MPa for 36 h showed early apoptosis with chromatin condensation, black structures along the nuclear membrane and membrane blebbing (*); (**c**) late apoptosis with nuclear fragments of formed apoptotic bodies and many intracellular vacuoles; and (**d**) the release of apoptotic bodies at the final stage of apoptosis. Magnification 8000×, scale bar: 2 μm.

TEM is considered the gold standard for confirming apoptosis. This is because categorization of an apoptotic cell is irrefutable if the cell contains certain ultrastructural morphological characteristics^13,21^. As shown in Fig. 3, the normal morphology of cells in the 0 MPa group (untreated) was distinct from the apoptotic cells in the 50 MPa group treated for 36 h. We noted electron-dense nuclei and many blebs (indicated by asterisk) at the cell surface in the early phase of apoptosis (Fig. 3b). In later phases, the cells still had intact membranes and fragmented into apoptotic bodies containing cytoplasmic organelles with or without nuclear fragments (Fig. 3c). At the final stage, the release of apoptotic bodies was noted (Fig. 3d).

### Involvement of reactive oxygen species (ROS) generation and caspase 3/7 in HHP treatment

**Figure 4:**
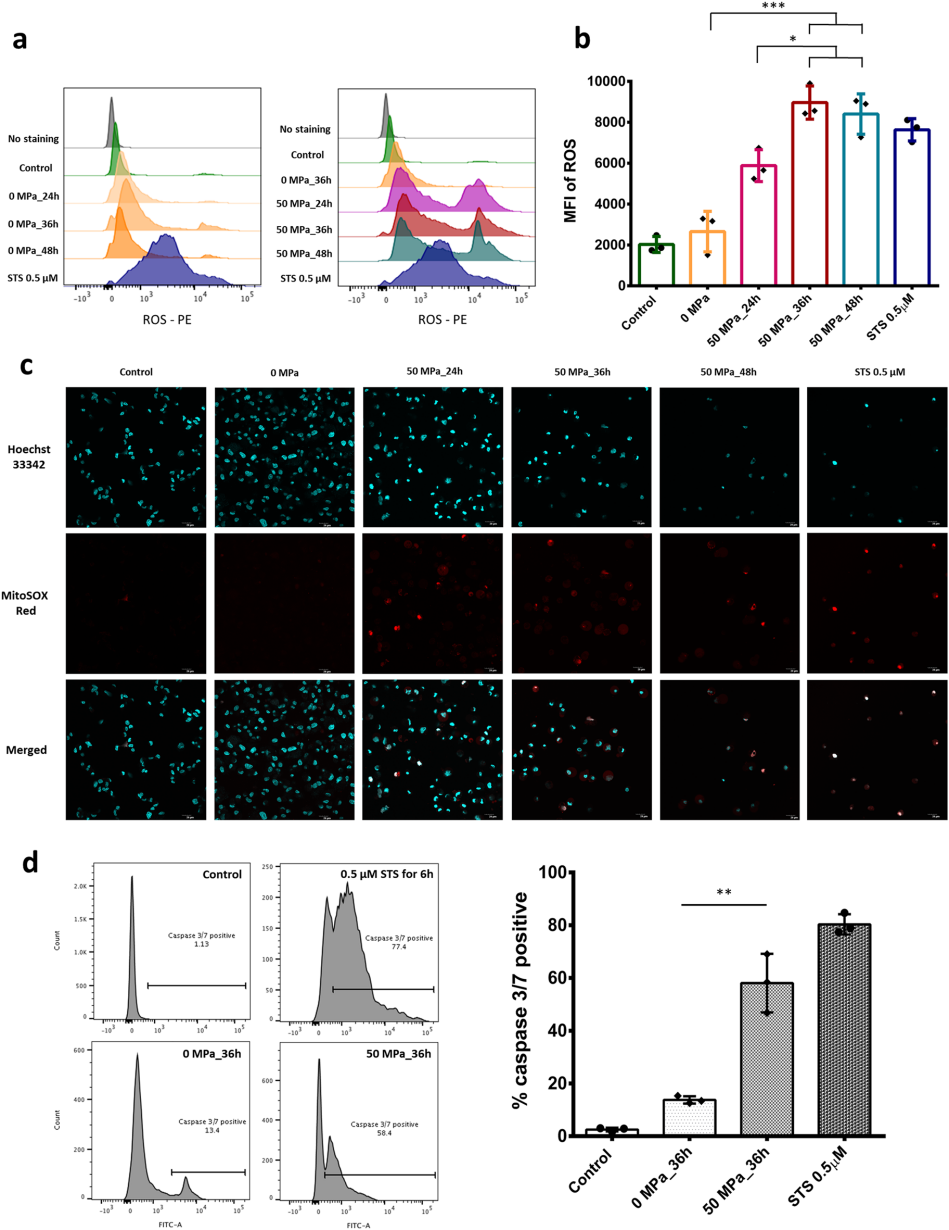
Reactive oxygen species (ROS) generation and activated caspase 3/7 in response to HHP treatment. (**a**) Left: representative histograms of the fluorescence expression of MitoSOX Red among the control, STS-treated and various time exposure of 0 MPa groups. Right: representative histograms of the fluorescence expression of MitoSOX Red among the control, STS-treated and HHP-exposed and unexposed groups. (**b**) Diagram comparing the MFI of ROS elevation among groups (n = 3). (**c**) Immunofluorescence images of ROS production stained by MitoSOX Red mitochondria indicator and Hoechst 33,342 showing the generation of a ROS signal after 50-MPa treatment for 24, 36 or 48 h. Scale bar: 20 μm. Data are representative of at least three independent experiments. (**d**) Activated caspase 3/7 fluorescence expression and ratio of caspase positivity between groups without HHP (0 MPa) and with 50 MPa for 36 h (n = 3).

ROS are implicated in the induction or enhancement of apoptosis^22–24^. We therefore used MitoSOX Red mitochondria indicator to demonstrate the overproduction of ROS in HHP-treated cells. In the present study, we used the 0 MPa group treated for 36 h as the reference for other exposure durations, as we wanted to elucidate the stark differences between the presence and absence of pressurization after 36 h. As shown on the left of Fig. 4a, there was no marked difference in the ROS expression among the control and 0 MPa groups (various durations). However, after 24 h of pressurization (right diagram), an increased ROS intensity was detected, with further increases observed over time. On comparing the ROS intensity (Fig. 4b), we noted substantial ROS production after HHP exposure at 50 MPa for 36 or 48 h and slight elevation after HHP exposure at 50 MPa for 24 h. Immunofluorescence images with MitoSOX Red and Hoechst 33342 counterstain showed a reduction in nuclear staining in the 50 MPa groups compared with the control and 0 MPa groups as well as the generation of MitoSOX Red signals in the groups with HHP treatment (Fig. 4c).

Caspases play a critical role in protein cleavage in the apoptotic pathway, with caspase-3 and caspase-7 considered the most important of the executioner caspases, being activated by other initiator caspases (caspase-8, caspase-9 or caspase-10)^17,25^. In our study, cells in warm closed bags (0 MPa) showed slightly higher ratios of activated caspase-3/7 than cells cultured at 37 °C in a 5% CO_2_ incubator, due to the environment not being optimal. However, after HHP exposure at 50 MPa for 36 h, cells showed high ratios of activated caspase-3/7 (60%), similar to STS treatment (Fig. 4d). A diagram comparison of the cells showed significantly higher levels of activated caspase-3/7 in the 50 MPa group than in the 0 MPa group after 36 h (p < 0.01).

### Viability and proliferation of HHP-treated cells

**Figure 5:**
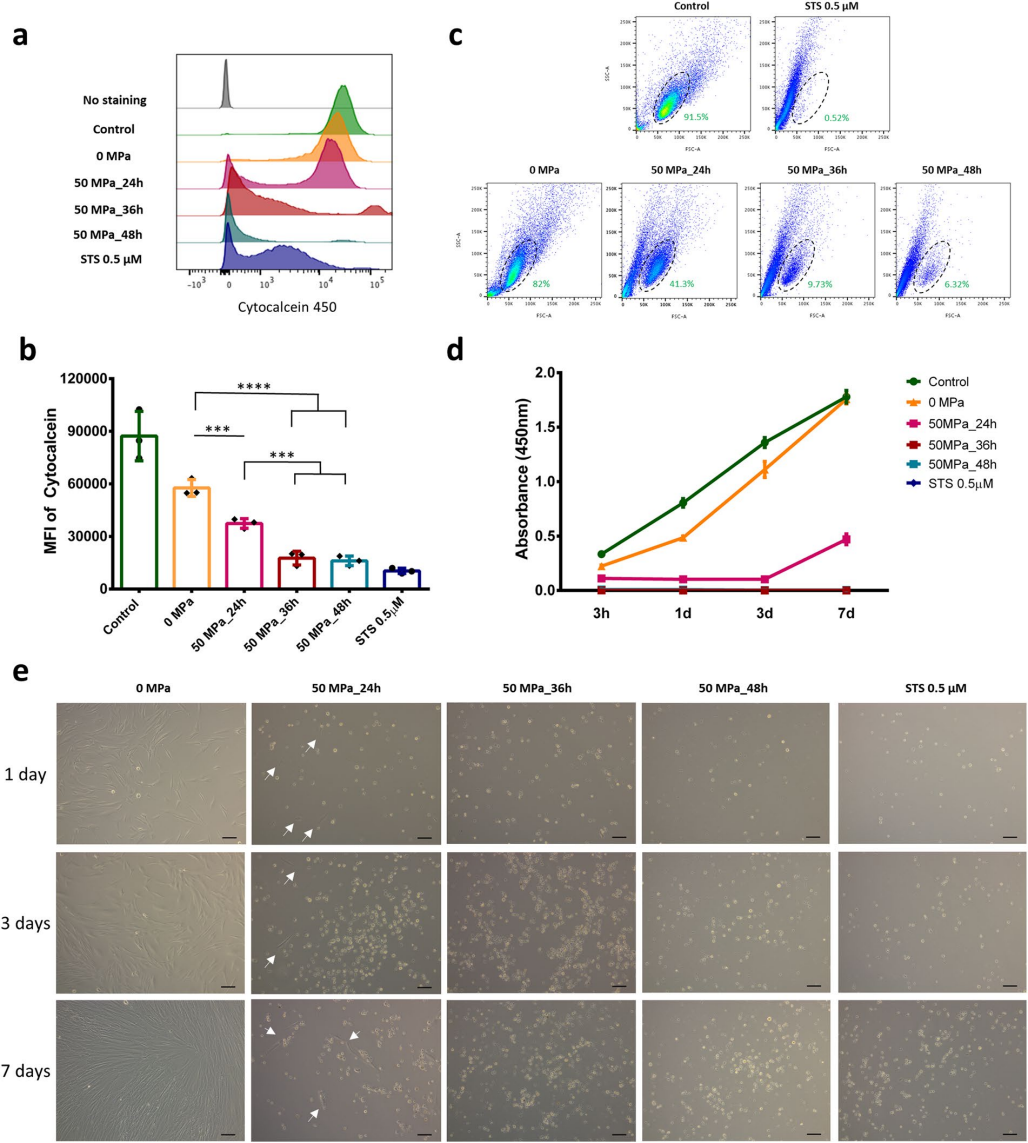
Viability and proliferation of HHP-treated cells. (**a**) Representative histograms of Cytocalcein 450 fluorescence (viability stained) among groups, with cells exposed to 50 MPa for 36 and 48 h showing negative expression, similar to STS treatment, while those pressurized for 24 h show bimodal distribution. (**b**) The comparison diagram shows a significant reduction in the Cytocalcein MFI between the 0 MPa group and the group treated with 50 MPa for 24, 36 or 48 h (n = 3). (**c**) A flow cytometry analysis showing two distinct cell populations depending on the cell size (FSC) and granularity (SSC). The oval area in the control group indicates normal viable cells, which are present in the positive control (STS) group at only 0.52%. That number in the 0 MPa group is around 82% while viable cells account for around 41.3% after being pressurized at 50 MPa for 24 h and 9.73% and 6.32% after 36 and 48 h, respectively. Data are representative of at least three independent experiments. (**d**) Absorbance at 450 nm in the WST-8 assay shows the proliferation of control, untreated, HHP-treated and STS-treated cells after 7 days of seeding culture. Data are representative of at least two independent experiments, n = 7 for each time point. (**e**) Inverted light microscopy images of untreated, HHP-treated and STS-treated cells seeded onto a 24-well plate after 7 days of culture. The white arrow in the 50 MPa_24h group indicates viable cells. Magnification 10×, scale bar 100 μm. Data are representative of at least three independent experiments.

As shown in Fig. 5a, the similarity of the histogram between the cells in the 0 MPa and control groups showed that culturing suspended fibroblasts in a closed bag for up to 36 h did not markedly affect the cell viability. After 24 h of pressurization, the Cytocalcein 450 intensity was reduced, and the presence of negative staining was evaluated. After 36 and 48 h of pressurization, the amount of negative staining was increased, and few positive-stained cells were detected. Figure 5b shows the slight reduction in the MFI from 0 to 50 MPa for 24 h, with a significant reduction noted after 36 and 48 h of pressurization (like STS). In order to confirm cell viability, the cell size (FSC) and granularity (SSC) according to a flow cytometer (without stained) were used to identify differences in the two populations (Fig. 5c). We noted that living cells, represented by a dot plot in the oval area, normally accounted for roughly 91.5% of the control group; this value was slightly lower in the 0 MPa group (82%) and then decreased under pressurization at 50 MPa over time (41.3%, 9.73% and 6.32% after 24, 36 and 48 h, respectively).

Another important point that must be evaluated concerning the complete induction of cell death is the possibility of death reversibility and proliferation after seeding to appropriate medium culture. Using a WST-8 assay and light microscopy, we found that the fibroblasts in the 0 MPa group were slightly weaker than those in the control group at 3 h after seeding but proliferated well subsequent, reaching confluency by day 7 (Fig. 5d,e). In contrast, the fibroblasts in the 50 MPa group treated for 24 h were weaker than those in the 0 MPa group and did not proliferate during the first 3 days, although they began proliferating again subsequently and continuously grew until day 7. Interestingly, in contrast to the findings shown in Fig. 5c, we did not detect any signals of viable cells after 50 MPa for 36 or 48 h, just as in the STS group, even after 7 days of seeding culture. These results indicate that 50 MPa for ≥ 36 h is effective for completely inducing irreversible cell death.

### Inducing apoptosis in a pressurized skin graft

**Figure 6:**
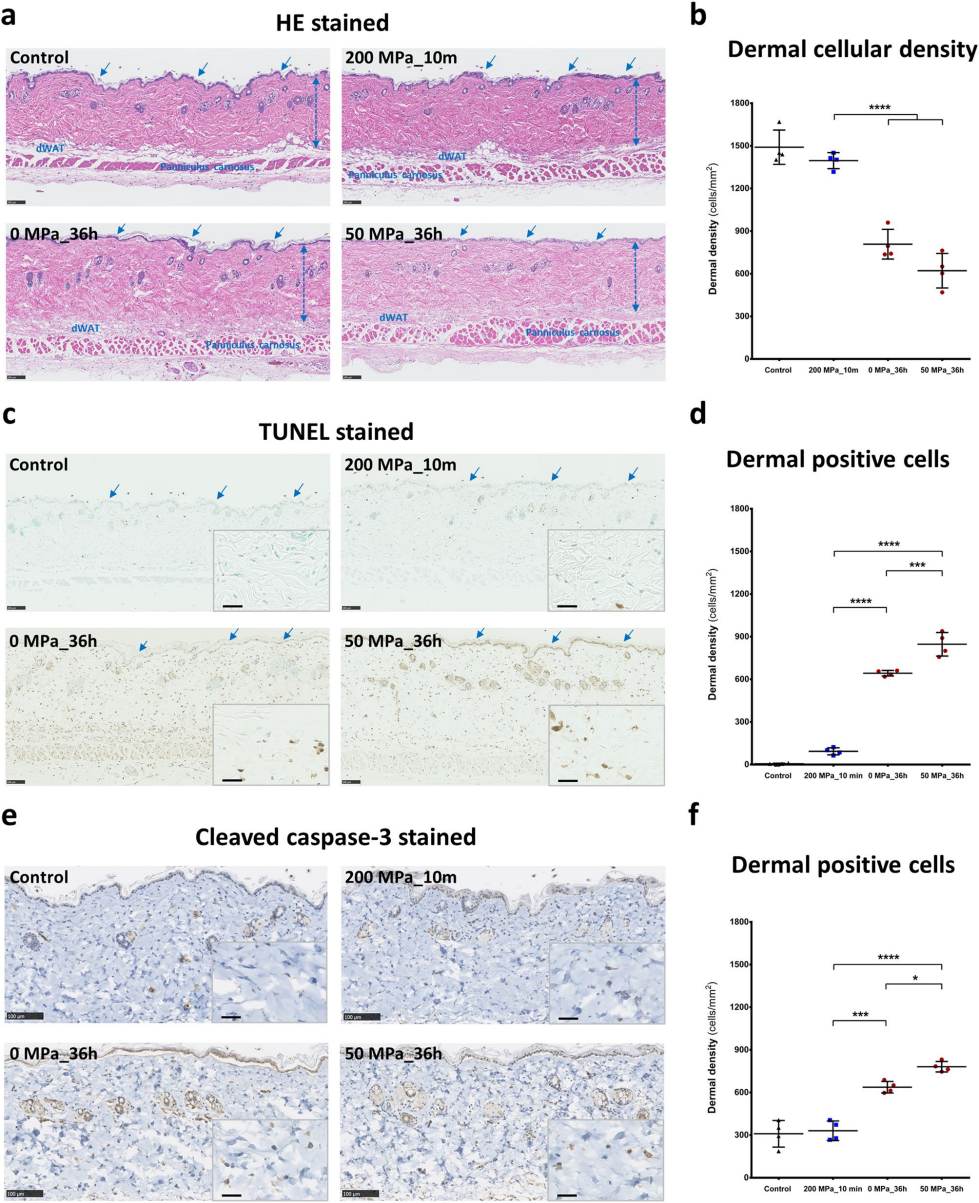
Ex vivo induced apoptosis in inactivated skin by HHP. (**a**) Representative HE stained micrographs of fresh mouse skin (Control), 0 MPa, 50 MPa, and 200 MPa. No marked differences were observed in the epidermis layer (blue arrow), dermal layer (blue dash arrow), collagen fibers, dermal white adipose tissue (dWAT), panniculus carnosus or other adnexal structures. Magnification 10×, scale bar 100 μm. (**b**) The diagram comparison of the dermal cellular density among groups shows a reduction in cell nuclei numbers in the 0 and 50 MPa groups after 36-h treatment (n = 4, p < 0.0001). (**c**) Representative TUNEL-stained micrographs of fresh mouse skin (Control) and skin exposed to 0, 50 and 200 MPa. Blue arrows indicate changes in the epidermis layer in the 0 and 50 MPa groups compared with the Control and 200 MPa groups. The immuno-positive signal intensity in the 0 and 50 MPa groups (brown color) was stronger than in the Control and 200 MPa groups. Magnification 10×, scale bar 100 μm. The higher-magnification micrographs in the lower right show more detail concerning each positively stained cell (brown signal) in all groups; scale bar 25 μm. (**d**) The diagram comparing the dermal TUNEL-positive cells among groups shows significant differences between the 50 MPa and other groups (n = 4, p < 0.0001 and p < 0.001). (**e**) Representative cleaved caspase-3-stained micrographs of fresh mouse skin (Control) and skin exposed to 0, 50 and 200 MPa. The brown-stained cells indicate immuno-positive signals. Magnification 20×, scale bar 100 μm. The higher-magnification micrographs in the lower right show more details concerning each positively stained cell (brown signal) in all groups; scale bar 25 μm. (**f**) The diagram comparing the dermal caspase-3-positive cells among groups shows differences between the 0 and 50 MPa groups and others (n = 4, p < 0.001) as well as a difference between the 0 and 50 MPa groups themselves (n = 4, p < 0.05).

Based on the results of the in vitro experiments above, we investigated the probability of skin tissue inactivation via apoptosis by HHP at 50 MPa for 36 h and compared the findings with the results obtained using the existing method. As shown in Fig. 6a, we observed no marked differences in the epidermis layer (blue arrow), dermal collagen or elastic fibers, dermal white adipose tissue (dWAT), panniculus carnosus or other adnexal structures among groups with fresh mouse skin (Control). However, we noted a reduction in the dermal cellular density of the 0 and 50 MPa groups treated for 36 h (Fig. 6b). This may be due to the spontaneously biodegradable of death cells in the dermis by this time. The TUNEL method is used to add a labeled UTP to the 3′-end of the DNA fragments and has been adopted as the method of choice for detecting apoptosis in situ due to its very sensitive staining^26^. In Fig. 6c,d, the micrograph of normal fresh skin (Control) shows almost no positive TUNEL staining, while a few positive signals were observed in the 200 MPa group (n = 4, p = 0.0664). In contrast, the high number of positive signals (brown color) in the micrographs of the 0 and 50 MPa groups visibly indicate the presence of many apoptotic cells (p < 0.0001). The blue arrows indicate the few changes in the epidermis layer in the 0 MPa group compared with the Control group, but the epidermis is completely apoptotic in the 50 MPa group. The numbers of positive dermal cells were also significantly higher in the 50 MPa group than in the 0 MPa group (p < 0.001). In addition, immunohistochemistry of cleaved caspase-3 antibody was used to confirm the appearance of apoptotic cells in those skin specimens (Fig. 6e,f). In the micrographs of anti-caspase-3 staining, we detected few positive cells (brown stained) in the 200 MPa group, but this was not markedly different from the natural apoptosis process in normal skin (n = 4, p = 0.9646). However, the numbers of positive cells in the 0 and 50 MPa micrographs were significantly increased (p < 0.001), and there were more positive cells in the 50 MPa group than in the 0 MPa group as well (p < 0.05).

In a previous study, Mahara et al. reported that HHP treatment at 200 MPa for 10 min completely killed NIH/3T3, smooth muscle and endothelial cells^5^. However, as shown in Fig. S1a concerning the mechanism underlying cell death, the proportions of TUNEL-positivity and anti-caspase-3 positivity were around 6.68% and 23.68%, respectively, of total cells. In contrast, the proportion of cell death by 50 MPa treatment was 136.26% for TUNEL and 125.75% for anti-caspase-3, indicating that our new method is effective for completely inducing cell death via apoptosis. Those results explain the difference between programmed cell death in 50 MPa specimens and the accidental cell death in 0 MPa specimens, however, this point was not the focus of our current study. We also assessed the viability of skin specimens after pressurization by the outgrowth culture method^8^. As shown in Fig. S1b, after 4 weeks in an appropriate medium culture, fibroblasts showed growth and proliferation from fresh skin and skin specimen of 0 MPa_36 h groups. In contrast, we did not observe any cells growth from the pressurized skin specimens of 50 or 200 MPa groups. In addition, the WST-8 absorbance levels reflecting the specimens’ viability in the 50 MPa_36 h and 200 MPa_10m groups were also 0 until 4 weeks (Fig. S1c).

### The in vivo infiltration of host cells to the inactivated dermis after implantation

**Figure 7:**
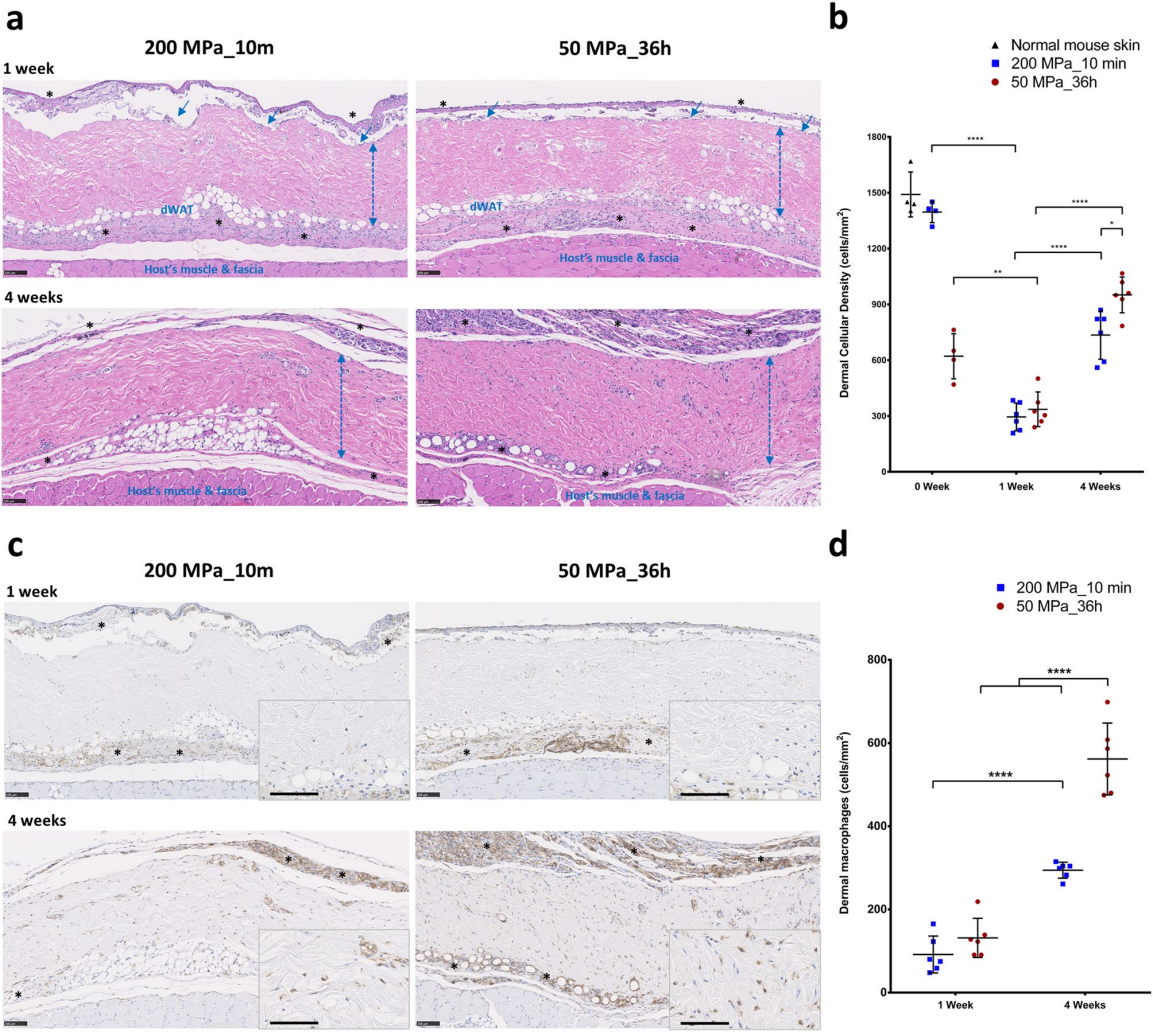
In vivo infiltration of host cells and macrophages to inactivated dermis. (**a**) Representative HE-stained micrographs of skin grafts treated by HHP at 200 MPa for 10 min or 50 MPa for 36 h in implantation. Magnification 10×, scale bar 100 μm, *: granulation tissue formation,  : dermis layer. (**b**) The diagram comparing the dermal cellular density shows a reduction in the numbers of cell nuclei in both groups at 1 week after implantation (n = 6, p < 0.01). At week 4, more cells had infiltrated into the inactivated dermis, followed by an increase in the dermal cellular density (p < 0.0001), and the density in the dermis of the 50 MPa group was higher than that is the 200 MPa group (p < 0.05). (**c**) Representative anti-F4/80 immunohistochemically stained micrographs of skin grafts treated by HHP at 200 MPa for 10 min or 50 MPa for 36 h in implantation. Magnification 10×, scale bar 100 μm, (*) indicates granulation tissue. The higher-magnification micrographs in the lower right show more detail concerning each positively stained cell (brown signal); scale bar 100 μm. (**d**) The diagram comparing the increase in numbers of infiltrated dermal macrophages at 4 weeks after implantation in both groups (n = 6, p < 0.0001) while the number is significantly higher in the grafts of the 50 MPa group than that in the 200 MPa group (p < 0.0001).

HE staining was used to explore the in vivo engraftment and regeneration of inactivated dermis (Fig. 7a). As mimicking the full thickness skin defect, we grafted the inactivated dermis on the subcutaneous of mice back, covered with collagen sheets and followed their regeneration. Therefore, in the histologic sections we could observe host’s muscle & fascia, inactivated skin grafts, but not host’s original skin. At 1 week after implantation, the epidermis layer (blue arrows) had been biodegraded and was completely discarded by 4 weeks. No severe inflammatory reaction or collagen fiber degeneration was observed in the dermal area as of 4 weeks after implantation. On comparing the histopathology of those grafts after pressurization (Fig. 6a), we observed a reduction in their dermal cellular density after 1 week of implantation. This may be due to the in vivo biodegradation or phagocytosis of original death cells in the inactivated dermis. Granulation tissue (indicated by asterisks) thereafter forms, and the cells infiltrate into the inactivated dermis in order to occupy and proliferate in the ECM. This phenomenon was clearly observed based on the increase in the dermal cellular density at 4 weeks after implantation. As shown in Fig. 7b, at week 1, the dermal cellular density in both groups was reduced (compared with the zero time point) and not significantly different (n = 6, p > 0.999). After 4 weeks, the dermal cellular density in both groups was increased (p < 0.0001), and a higher density was found in the skin grafts of the 50 MPa group (p < 0.05). Furthermore, anti-F4/80 immunohistochemistry staining was used to detect macrophage activity in the inactivated dermis (Fig. 7c). At week 1, we noted the accumulation of macrophages in the subcutaneous layer (indicated by asterisks) and above the epidermis layer, but only few macrophages were observed in the dermis. We consider those cells to play a role in the phagocytosis of cell debris and granulation tissue formation. At week 4, the granulation tissue was increased, and a large number of macrophages had infiltrated into the inactivated dermis, especially in the 50 MPa group. The comparison of the dermal macrophage density in Fig. 7d showed no marked difference between the groups at week 1 (n = 6, p = 0.775), but the density was elevated at 4 weeks after implantation (p < 0.0001) and it was much higher in the skin grafts of the 50 MPa group (p < 0.0001).

### Newly formed capillaries of the inactivated dermis after implantation

**Figure 8:**
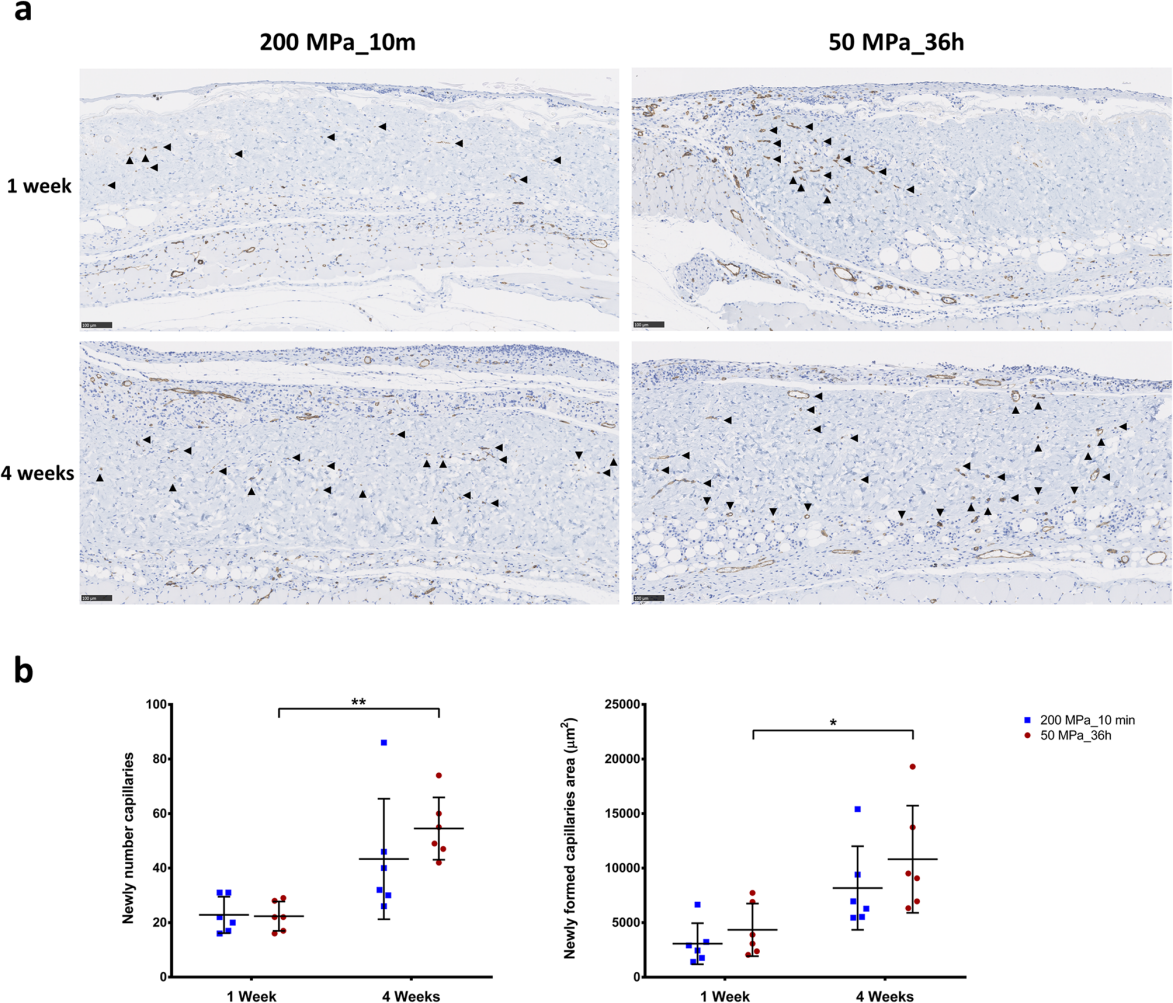
Newly formed capillaries of inactivated dermis in implantation. (**a**) Representative anti-CD31 immunohistochemically stained micrographs of skin grafts treated by HHP at 200 MPa for 10 min or 50 MPa for 36 h after implantation. New capillaries were formed in both groups from week 1 and then increased in size and number by 4 weeks after implantation. Magnification 10×, scale bar 100 μm, filled black triangle : newly formed capillary. (**b**) The diagram comparing the number of newly capillary formations shows elevated values in both groups after 4 weeks, although only to a significant degree in the 50 MPa group (n = 6, p < 0.01). Similarly, after 4 weeks, the area of newly formed capillaries had increased as well but only to a significant degree in the 50 MP group (p < 0.05).

Micrographs of the anti-CD31 immunohistochemical staining of grafts after 1 and 4 weeks are shown in Fig. 8a. This staining detected capillaries (black arrowhead) in the grafts of both groups, thus revealing the blood flow to the grafts. Only a few small capillaries were observed at week 1 in both groups. However, the capillary formation increased in number and size at 4 weeks after implantation. In the comparison of newly formed capillaries number and area (Fig. 8b), although no marked differences between the 200 and 50 MPa groups were noted at each time point, we observed a significant increase in both the newly formed capillaries number (p < 0.01) and capillaries area (p < 0.05) in the 50 MPa group over this time, while no such changes were noted in the grafts of the 200 MPa group.

## Discussion

In a number of HHP-related experiments, researchers showed that mammalian cells die when treated with pressures around 200 MPa, while treatment with HHP < 150 MPa did not markedly affect the viability of human cells^27–29^. We previously confirmed that HHP at 200 MPa for 10 min was able to inactivate mammalian cells, skin or melanocytic nevus tissue; however, inactivation was not noted at HHP < 150 MPa^5,30,31^. In contrast to the above method involving high-pressure treatment for a short duration of 10 min, which causes cell membrane rupture followed by necrosis or necroptosis, and thus may be responsible for immune rejection in grafting^32^ or result in scar formation, we reduced the pressure magnitude to 50 MPa and attempted long-term exposure in order to induce cell death mainly via apoptosis. We consider this mechanism to be critical for preventing inflammation of the inactivated tissue after transplantation. Instead, apoptosis cell-death and apoptotic bodies, which expose eat-me signals, are engulfed by host macrophages or dendritic cells, thereby avoiding promoting any inflammatory reaction^33,34^. A few previous studies reported the induction of chondrocyte apoptosis by in vitro persistent HHP treatment, as in our current study^35,36^. They found that the cell viability was reduced after pressurization but not completely eliminated, as a few cells remained alive which may promote immune or inflammatory reaction after transplantation.

As the primary study using HHP at 50 MPa for completely inducing cells to undergo apoptosis; firstly, we investigated the specific time exposure to reach that effect. We attempted to treat fibroblasts by HHP at 50 MPa (each of 0 MPa was used as approriate reference) for 6 h, 9 h, 18 h, 24 h, 36 h, 48 h, 120 h, and even for 1 week. Based on those results, we found that 50 MPa treatment for 36 h is sufficient to completely induce cell death by apoptosis, while no significant differences of apoptotic cells among 0 MPa for 24 h, 36 h or 48 h treatments (Fig. S2a). Hence, we used fibroblasts treated by HHP at 0 MPa for 36 h as a representative reference for 0 MPa group in all data for the in vitro experiments. To prove the presence of distinct differences, we performed the experiments again, using cells treated with 50 MPa pressure for 24 h, 36 h, 48 h in one process together with 0 MPa for 36 h as representative for the 0 MPa group (our HHP device is limited to 4 isostatic chambers at present) and these results are shown in Figs. 2, 4, 5. In addition, we also investigated the impact of lower pressures, such as 10, 20 and 30 MPa for 48 h in order to confirm the cut-off level of HHP treatment (Fig. S2b–d). We found that HHP at 10 MPa had no effect of inducing apoptosis compared with 0 MPa; while HHP at 20 MPa induced cell death incompletely, with around 30% of cells going apoptotic compared with more than 90% of cells going apoptotic in the 30 and 50 MPa groups. Those results indicated that HHP treatment from 10 to 50 MPa for long time is able to induce apoptosis, but its effects depend on the pressure magnitude and duration of exposure.

In the manufacture of the HHP device, traditional HHP devices generally have only one isostatic chamber due to complicated stabilization management. Thus, it has been difficult until now to extend the application of HHP techniques in the field of medical science. By reducing the pressure magnitude to around 50 MPa, we can expand more independent processing by increasing the number of isostatic chambers. Since our newly developed HHP device has four separate chambers with the possibility for further expansion, we were able to easily include various pressure levels and time exposures in our experiment. In general, this is extremely helpful for the processing of a large amount of samples, thereby increasing the cost effectiveness of this technique.

To demonstrate the effect of HHP on cell viability, we performed a quantitative evaluation of the cells after HHP at 50 MPa using a WST-8 assay, which is a sensitive colorimetric assay for determining the number of viable cells in proliferation and cytotoxicity^37,38^. The results in Fig. 5d indicated that the fibroblasts became completely inactivated at 50 MPa for 36 or 48 h, in contrast to the small percentage of cells emitting a blue color (cytocalcien450-positive, indicating viability) in apoptosis/necrosis staining (Fig. 2e) or those that were present in the live population (Fig. 5c). In our previous study, we showed that the cellular esterase activity persisted for several hours after HHP treatment, even with ultra-high pressurization at 500 MPa^5^. This may explain why we failed to observe any living cells after exposure to 50 MPa for 36 or 48 h for up to 7 days’ culture under a light microscopy (Fig. 5e).

In the in vivo study, we focused on the reformation of inactivated dermis after implantation. We previously reported that HHP treatment exceeding 200 MPa for 10 min was sufficient to induce cell death through the inactivation of mitochondrial activity, resulting in the complete inactivation of human skin, human nevus specimens and porcine skin without damaging the structure of ECM^5,30,31^. In those studies, we found that the original cells were removed after several days of implantation, then host cells including but not limited to fibroblasts, macrophages, endothelial cells infiltrated to the remained ECM of the inactivated dermis, proliferated there, and contributed to dermis regeneration. Eming et al. reported that the tissue formation phase of wound healing was characterized by dynamic cellular proliferation and differentiation of cells along with the creation of new ECM and deposition of collagen to support the new cells^39^. During that phase, macrophages are considered to play a critical role in efficient wound healing because they contribute to all aspects of this process of cell growth, matrix deposition and controlled inflammation. As shown in Fig. 7c, we observed the appearance of macrophages at week 1 which played role for clearing the original inactivated cells, then macrophage numbers increased alongside with increasing of other cells or capillaries formation in the dermis from week 1 to week 4, following the increasing of neovascularization in the inactivated dermis (Fig. 8). Therefore, we consider those are not signs of chronic inflammation, and infiltrated macrophages can contribute to accelerate tissue regeneration.

Furthermore, macrophages are also major sources of growth factors in the skin. Macrophage-derived growth factors induce not only differentiation and proliferation of cells but also ECM deposition^40^. As shown in Fig. 7, the skin grafts of the 50 MPa group, which were inactivated by apoptosis, attracted more cells and showed a greater accumulation of macrophages in the dermis than skin grafts of 200 MPa group. Given this increased density of macrophages in the dermis at 4 weeks after implantation, we believe that the tissue re-formation of grafts in the 50 MPa group is more effective than that of grafts in the 200 MPa group. These data suggest that skin inactivated by HHP at 50 MPa can be grafted without removing the cellular remnants that have been reported to induce inflammation.

## Materials and methods

### Study design and ethical standards

The present study included three parts: an in vitro investigation of the potential to induce cell death of the fibroblast cell line completely via apoptosis, an ex vivo attempt to inactivate skin tissue using the selected pressurization conditions and the in vivo implantation of that inactivated skin graft. Animal experimental procedures were conducted in accordance with the Guidelines for Animal Experiments established by the Ministry of Health, Labor and Welfare of Japan and were approved by the ethics committee of Kansai Medical University (permit No. 17-002(02)).

### Newly developed automatic physiological HHP device

We jointly developed an automatic HHP device in collaboration with Echigo Seika Co., Ltd. (Nagaoka, Japan). This system is an innovative, complex pressure instrument that consists of four separate main pressure units with pressure generation (hydraulic pumping), a circulating liquid temperature control system and a computerized pressure control unit (Fig. 1c). The dimensions of each isostatic chamber are ϕ52 mm × 150 mm with an inner volume capable of holding approximately 300 ml. The temperature management system consists of 2 units with a large adjustment range (− 30 to 95 °C) for each of the 4 isostatic chambers. In the current study, the applied temperature was maintained at 36.5 ± 0.5 °C inside all chambers for long-term pressurization. From the touch screen control center, the pressurization process is simplified, and operators can easily set the pressure (10–60 MPa) and treatment duration for each chamber. They also can observe the real-time pressure magnitude, temperature and count-down time of pressurization from this screen (Fig. 1c). The computerized control unit is always active while processing to ensure the pressure inside the chambers stays within ± 1 MPa and then automatically reduces the pressure to 0 when the session is finished. The control unit can connect to other systems via a local area network (LAN) and record the pressurization according to the requirements of the clinical trial.

### Inducing apoptosis of human dermal fibroblasts

Human dermal fibroblast adult (hDFa; Catalog number: C0135C; Thermo Fisher Scientific Inc., Waltham, MA, USA) cells were cultured using Dulbecco’s modified Eagle’s medium (DMEM; “Nissui”1; Nissui Pharmaceutical Co., Ltd., Tokyo, Japan) supplemented with 10% fetal bovine serum (FBS; Hyclone, Logan, UT, USA) and 1% antibiotic/penicillin and streptomycin solution (MP Biomedicals, LLC, Solon, OH, USA) at 37 °C, 95% humidity, and 5% CO_2_. After rapidly thawing in a water bath, 1 × 10^6^ cells were seeded and cultured on a 10- or 15-cm culture dish (Falcon; Corning Inc., Corning, NY, USA), after which the medium was changed every 1 or 2 days. When cell confluency reached 70–80%, they were washed 3 times with PBS and then dissociated using TrypLE Express (Gibco, Thermo Fisher Scientific Inc.). A total of 1 × 10^6^ cells in suspension were then dissolved in a sterile plastic bag filled with 50 ml culture medium, which was subsequently sealed. Cells at passages 6–12 were used in the experiment.

We established 4 pressurization conditions in this study: 0 MPa for 36 h and 50 MPa for 24, 36 or 48 h. In brief, when a stable temperature of 36–37 °C was reached in all isostatic chambers, a sealed bag was placed into each chamber, which was then filled with warm tap water and closed tightly. The pressure was then increased to 51 MPa in chambers 1-2-3, while no pressure was applied in chamber 4 (0 MPa, Fig. 1c), and the countdown was started until 0 was reached, at which point the pressure was reduced automatically by the system. The same passages of fibroblasts were cultured in a 10-cm culture dish at 37 °C in a 5% CO_2_ incubator as control cells, while adherent fibroblasts treated with 0.5 μM Staurosporine (STS; ab120056; Abcam Co., Ltd., Boston, MA, USA) for 24 h were used as a positive control^41,42^.

### The flow cytometric analysis of apoptotic cells treated by HHP

Immediately after pressurization, 3 × 10^5^ cells per sample were collected and washed in PBS. For the detection of phosphatidylserine (PS) membrane translocation, the cells were then resuspended in a mixture of 200 μl Assay Buffer, 2 μl Apopxin Green Indicator, 1 μl 7-AAD and 1 μl CytoCalcein 450 (Apoptosis/Necrosis Detection Kit; ab176749; Abcam Co., Ltd.). The samples were kept in the dark at room temperature (RT) for 30–60 min, and 2 × 10^4^ cells were recorded for the subsequent analysis on BD FACSCanto II (BD Biosciences, San Jose, CA, USA) using the Flowjo software program (Flowjo LLC, BD Biosciences). The total numbers of apoptotic cells (Apopxin^+^/7-AAD^+^ and Apopxin^+^/7-AAD^−^) were analyzed and compared.

The overproduction of reactive oxygen species (ROS) in the apoptotic cells was evaluated using MitoSOX Red Mitochondrial Superoxide Indicator (Molecular Probes, Thermo Fisher Scientific Inc.). First, 1 μl of 5 mM MitoSOX reagent stock solution was diluted in 1 ml of HBSS/Ca/Mg (Gibco, Thermo Fisher Scientific Inc.) and then added to cell pellets for each sample. Those samples were incubated at RT in the dark for 30 min and then analyzed using BD FACSCanto II. In addition, after HHP exposure, 1 μl of CellEvent Caspase-3/7 Green Detection Reagent (CellEvent Caspase-3/7 Green Flow Cytometry Assay Kit; Thermo Fisher Scientific Inc.) was added to 1 ml of medium containing approximately 3 × 10^5^ cells per sample and incubated for 60 min at RT, protected from light. During the final 5 min of staining, 1 μl of 1 mM SYTOX AADvanced dead cell stain solution (CellEvent Caspase-3/7 Green Flow Cytometry Assay Kit; Thermo Fisher Scientific Inc.) was added, and then 2 × 10^4^ cells were recorded for a subsequent analysis on BD FACSCanto II (BD Biosciences) using the Flowjo software program (Flowjo LLC, BD Biosciences).

### Immunofluorescence observation

After HHP treatment, 1 × 10^5^ cells per sample were washed with PBS, resuspended in fresh DMEM/ 10% FBS, and seeded in a 35-mm glass-bottom dish (Matsunami Glass Ind., Ltd., Osaka, Japan), after which they were stored for 30–60 min at 37 °C in a 5% CO_2_ incubator to encourage attachment to the dish bottom. For apoptosis/necrosis observation, the medium was gently discarded, and cells were washed 2 times with 200 μl of Assay Buffer. The cells attached to each glass-bottom dish were then incubated with a mixture of 200 μl Assay Buffer, 2 μl Apopxin Green Indicator, 1 μl 7-AAD and 1 μl CytoCalcein 450 (ab176749; Abcam) at RT for 30–60 min. For imaging ROS production, 1 ml of 5 μM MitoSOX reagent working solution (Molecular Probes) was administered to each glass-bottom dish, which was then incubated at 37 °C for 10 min. After gently washing 3 times with PBS, those cells were counterstained with Hoechst 33342 solution (Dojindo Molecular Technologies, Inc., Kumamoto, Japan) and incubated at RT for 15 min. Finally, images were acquired using a confocal laser scanning microscope (Fluoview FV3000; Olympus Co., Tokyo, Japan).

### Detection of apoptotic cells by transmission electron microscopy (TEM)

Upon completion of several passages after thawing, adherent fibroblasts were dissociated and packed into plastic bags for pressurization. Only the cells pressurized at 0 and 50 MPa for 36 h were observed. Immediately after HHP, the cells were collected by centrifugation and washed twice in PBS. The specimens for TEM observation were prepared following the protocol as described in our previous study^12^. Briefly, pressurized or non-pressurized cells were then fixed with fixative solution consisting of 2% glutaraldehyde, 0.1 M sodium cacodylate and 1 mM CaCl_2_ at 37 °C, pH 7.4 for 30 min; then washed 2 times with cacodylate buffer (0.1 M sodium cacodylate and 0.2 M sucrose) at 4 °C, pH 7.4 for 10 min in order to stop fixation. Next, samples were post-fixed with 1% osmium tetroxide (OsO_4_) in cacodylate buffer at 4 °C, pH 7.4 for 30 min and then dehydrated by ethanol. After that, they were infiltrated in Epon 812 resin and then polymerized at 45 °C for 12 h, 55 °C for 24 h and 45 °C for 12 h. Lastly, the specimens were cut into ultrathin sections and observed by TEM (JEM-1400Plus; JEOL Ltd., Tokyo, Japan).

### Morphology and viability of cells after pressurization

In brief, after HHP treatment, all of the medium (including cells) in each bag was harvested and then transferred to 50-ml conical tubes (Falcon; Corning Inc.). The tubes were centrifuged at 1600 rpm for 3 min, then supernatant was discarded, and the cell pellets were resuspended in 1 ml of fresh medium to count the number of cells remaining. As in the detection of the appearance of fibroblast aggregations in the 0 MPa group, the cell pellets were washed twice with PBS and then detached by 3-min exposure to 0.5 ml of TrypLE Express in the incubator. Due to the differences in the cell populations among groups, FSC and SSC of flow cytometry were used to assess the viability of cells based on the cell size and granularity, respectively. Furthermore, the staining of CytoCalcein 450 in the Apoptosis/Necrosis Detection Kit (ab176749; Abcam) specifically differentiated live cells from apoptotic or necrotic cells in the whole population. Therefore, the MFI of that staining modality was analyzed as well in order to compare the survival among treated cells.

To confirm the irreversibility of cell death after HHP treatment, a 100-µL aliquot of each cell suspension (adequation 1 × 10^4^ cells), including the control and STS groups, was seeded into a 24-well cell culture plate (Falcon; Corning Inc.) with 1 ml of the culture medium, and followed up to 7 days without changing medium in 37 °C/ 5% CO_2_ incubator. Then, cultured fibroblasts morphology and proliferation were observed and examined by an inverted microscope (Carl Zeiss Co., Ltd, Oberkochen, Germany) after 3 h, 1 day, 3 days and 7 days.

Furthermore, the WST-8 (Colorimetric Cell Viabilty) assay was used to quantitatively evaluate the proliferation of those cells. After pressurization, a 100-µL aliquot of each cell suspension (approximately 1 × 10^4^ cells, n = 7 per group) was added to four 96-well plates (Falcon; Corning Inc.), then incubated at 37 °C/ 5% CO_2_ incubator for 3 h, 1 day, 3 days or 7 days without changing the medium. At each evaluation time point, 10 µL of Cell Counting Kit-8 (CCK-8; Dojindo, Inc.) was added to each well and incubated at 37 °C for 2 h following manual instructions. Finally, the absorbance of medium was determined at a wavelength of 450 nm using an EnSpire Multimode Plate Reader (PerkinElmer Co., Ltd., Waltham, MA, USA). The absorbance of medium alone was used as blank (n = 7).

### Preparation and in vivo implantation of inactivated murine skin grafts

Briefly, C57BL6J/Jcl mice (CLEA Japan Inc., Tokyo, Japan) were euthanized by carbon dioxide inhalation and their skin backs were resected. Then, 8-mm punch biopsy tools (Kai Industries Ltd., Gifu, Japan) were used to obtain full-thickness skin grafts (total of 80 skin grafts). Ten grafts were packed into each of 25-ml cryopreservation bottles (Perfluoroalkoxy [PFA] bottle; Tech-jam, Osaka, Japan) filled with DMEM solution (Nissui Pharmaceutical Co.). Skin grafts in each bottle were pressurized at 50 MPa for 36 h using a new automated HHP device as mentioned above or at 200 MPa for 10 min using another custom-made device (Echigo Seika Co.), as described in our previous studies^11,30^ (Fig. 1b). After pressurization, those specimens were washed 2 times with PBS and transplanted to the dorsal fascia of other mice (2 grafts per mouse, total 12 grafts per group). We then covered the grafts with collagen sheets and closed the skin by nylon 5-0 sutures (Fig. 1b).

### The sampling and histological evaluation of the skin grafts

After pressurization, skin specimens were collected and fixed with 10% formalin-buffered solution (Wako Pure Chemical Industries, Ltd.) and then embedded in paraffin blocks. Immunohistochemical staining for terminal deoxynucleotidyl transferase dUTP nick-end labeling (TUNEL) and cleaved caspase-3 were performed for investigation of apoptotic skin, while Hematoxyline-Eosin (HE) staining was used to confirm the changes of the skin structure. In in vivo experiment, 1 and 4 weeks after implantation, three mice per group were euthanized using carbon dioxide inhalation. The implanted grafts (n = 6 per group) were harvested and fixed with 10% formalin-buffered solution (Wako Pure Chemical Industries, Ltd.). Paraffin Sects. 5-μm-thick of each specimen were subjected to HE staining and immunohistochemical staining for F4/80 and anti-CD31. Finally, histological sections were examined by NanoZoomer 2.0 HT imaging (Hamamatsu Photonics, Hamamatsu, Japan) at 40× magnification and analyzed using the NDP.view2 software program (Hamamatsu Photonics). Cell nuclei in 3 areas measuring 250 × 250 μm (at the center and on both sides 500 μm from the center) on HE sections at 1 and 4 weeks after implantation were counted and compared.

### The immunohistochemical evaluation of the tissue in situ apoptosis and the newly formed capillaries as well as macrophage inflammation in vivo

Firstly, the histology sections were cut in 5 μm slices, deparaffinized and rehydrated. A heat-induced epitope retrieval method was performed using Retrieval solution (pH 9; Nichirei Biosciences Inc., Tokyo, Japan) and incubated at 98 °C for 20 min (anti-CD31, anti-Caspase-3). For anti F4/80, sections were pre-heated with 10 mM sodium citrate buffer (pH 6.0; Sigma-Aldrich Japan. Co. Ltd., Tokyo, Japan) and incubated at 98 °C for 40 min. After that, all sections were immersed in 0.3% hydrogen peroxide (H_2_O_2_; Wako Pure Chemical Industries, Ltd.) for 10 min to block endogenous peroxidase activity. Next, the tissues were treated with protein blocking solution (3% bovine serum albumin in PBS; Sigma-Aldrich Japan Co., Ltd.) for 60 min and incubated in the first antibodies anti-CD31 (1:1000; ab182981), anti Capase-3 (1:250; ab184787) or anti F4/80 (1:2000; ab6640) at 4 °C overnight. Then, the peroxidase-labeled secondary antibody (Histofine Simple Stain MAX PO; Nichirei Biosciences Inc.) was applied at room temperature for 60 min. Finally, the immunohistochemical reaction was developed with 3,3′-diaminobenzidine tetrahydrochloride (DAB; Nichirei Biosciences Inc.), counterstained with hematoxylin and mounted. For TUNEL detection, paraffin sections were stained using ApopTag Peroxidase In Situ Apoptosis Detection Kit (S7100; Merk Milipore Ltd., Burlington, MA, USA) according manual instructions.

In the immunohistochemical sections for TUNEL, cleaved caspase-3 and F4/80 macrophages, typically positive cells in 3 areas measuring 250 × 250 μm (at the center and on both sides 500 μm from the center) were counted in each section, and the dermal density was calculated and compared. In the anti-CD31 immunohistochemical sections, the newly formed capillaries with a clearly visible lumen in all the dermal part were determined manually using the NDP.view2 software program, and their sum was used for the data analysis.

### Statistical analyses

The results are shown as the mean values of at least three independent experiments and standard deviation (SD), represented by bars. The significant of differences was estimated by Student’s *t*-test or a one-way analysis of variance (ANOVA), followed by the Tukey–Kramer post hoc test using the Prism software program, ver. 7.03 (GraphPad Software, Inc., San Diego, CA, USA). Values of *p < 0.05, **p < 0.01, ***p < 0.001, ****p < 0.0001 represent the level of significance (p < 0.05 was considered significant).

## Supplementary information

Supplementary Figure Legends.

Supplementary Figure S1.

Supplementary Figure S2.

## Data Availability

All data generated or analysed during this study are included in this published article.
