# Supplementary material for: Hydrostatic pressure can induce apoptosis of the skin
Source: Sci Rep. 2020 Oct 19;10:17594. doi: 10.1038/s41598-020-74695-5 (PMC7572420; doi:10.1038/s41598-020-74695-5)
Supplement: Supplementary file 1 — Supplementary Figure Legends. [file 41598_2020_74695_MOESM1_ESM.docx]

**Hydrostatic pressure can induce apoptosis of the skin**

(Supplementary Information)

Tien Minh Le^1†^, Naoki Morimoto^1,2*^, Nhung Thi My Ly^3†^, Toshihito Mitsui^1^, Sharon Claudia Notodihardjo^1^, Maria Chiara Munisso^2^, Natsuko Kakudo^1^, Hiroyuki Moriyama^4^, Tetsuji Yamaoka^5^, Kenji Kusumoto^1^

^1^ Department of Plastic and Reconstructive Surgery, Kansai Medical University, Hirakata, Osaka, Japan

^2^ Department of Plastic and Reconstructive Surgery, Graduate School of Medicine, Kyoto University, Kyoto, Japan

^3^ Department of Dermatology, Kansai Medical University, Hirakata, Osaka, Japan

^4^ Pharmaceutical Research and Technology Institute, Kindai University, Higashi-Osaka, Osaka, Japan

^5^ Department of Biomedical Engineering, National Cerebral and Cardiovascular Center

Research Institute, Suita, Osaka, Japan

**†** Both authors contributed equally to this manuscript

* Corresponding author

E-mail: mnaoki22@kuhp.kyoto-u.ac.jp

**Figure S1. Exploring cell death in the pressurized skin grafts. (a)** A summary of the cell death mechanism of HHP-treated skin specimens shows that the cell death in the 50 MPa group was largely apoptotic (n=4, * p<0.05, *** p<0.001, **** p<0.0001). **(b)** Inverted light microscopy images of an untreated skin specimen (Control), without pressurization (0 MPa_36 h) and with pressurization (50 MPa or 200 MPa) cultured in 24-well plates. By 4 weeks later, fibroblasts had appeared and proliferated in the Control and 0 MPa groups; in contrast, non-viable cells were observed in the HHP-treated groups. Magnification 10x, scale bar 250 μm, asterisks indicate the skin specimen area. **(c)** Absorbance at 450 nm in the WST-8 assay indicates the proliferation of control, untreated, and HHP-treated skin specimens after 4 weeks of outgrowth culture (n=3 for each time point). Data are representative of three independent experiments.

**Figure S2. Investigating the effects of HHP on fibroblasts. (a)** A representative FCM analysis comparison between 0 MPa and 50 MPa at each specific time exposure for 24h, 36h, 48h, 120h, and 1 week indicate that survival of fibroblasts in 0 MPa for 24h, 36h, 48h are not different while their survival significantly reduce after 120h or 1 week treatment. **(b)** A representative FCM analysis of untreated and HHP-treated cells at different pressures after 48 h, showing similarities between the untreated group and the groups exposed to 10 and 20 MPa. HHP treatment at 30 MPa induced cell death similar to 50 MPa after 48 h. **(c)** The comparison of the apoptotic cell ratio indicated significant differences between the 30-50 MPa groups and the lower pressure groups (n=3). **(d)** Inverted light microscopy images of untreated and HHP-treated cells seeded onto 24-well plates after 7 days. Fibroblasts in the 30 and 50 MPa groups showed non-viable morphologies and an inability to recover or proliferate afterwards. Magnification 10x, scale bar 100 μm. Data are representative of at least three independent experiments.
