# Supplementary figures and images for: Hydrostatic pressure can induce apoptosis of the skin
Source: Sci Rep. 2020 Oct 19;10:17594. doi: 10.1038/s41598-020-74695-5 (PMC7572420; doi:10.1038/s41598-020-74695-5)

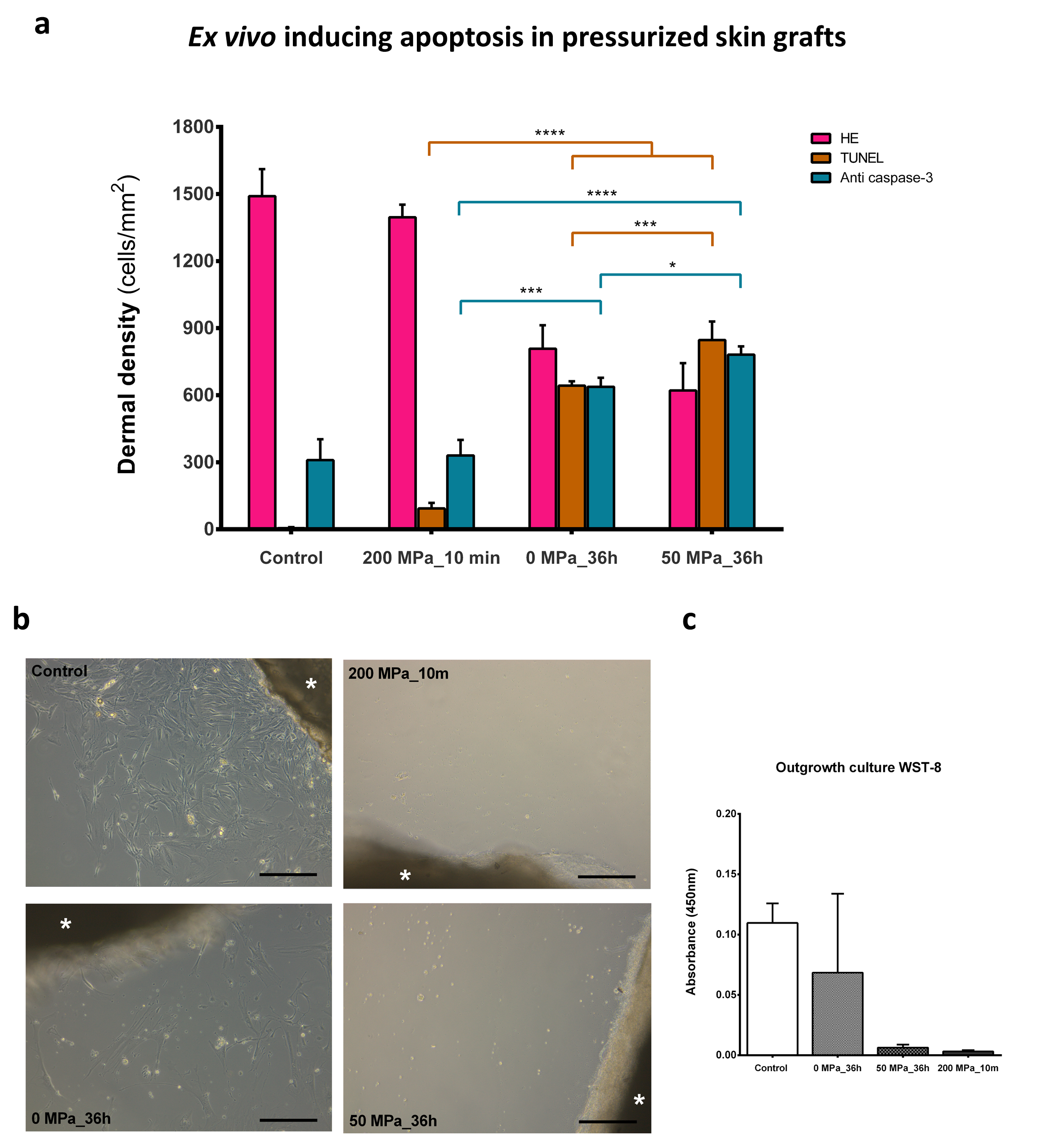

Supplement: Supplementary file 2 — Supplementary Figure S1. [file 41598_2020_74695_MOESM2_ESM.tif]

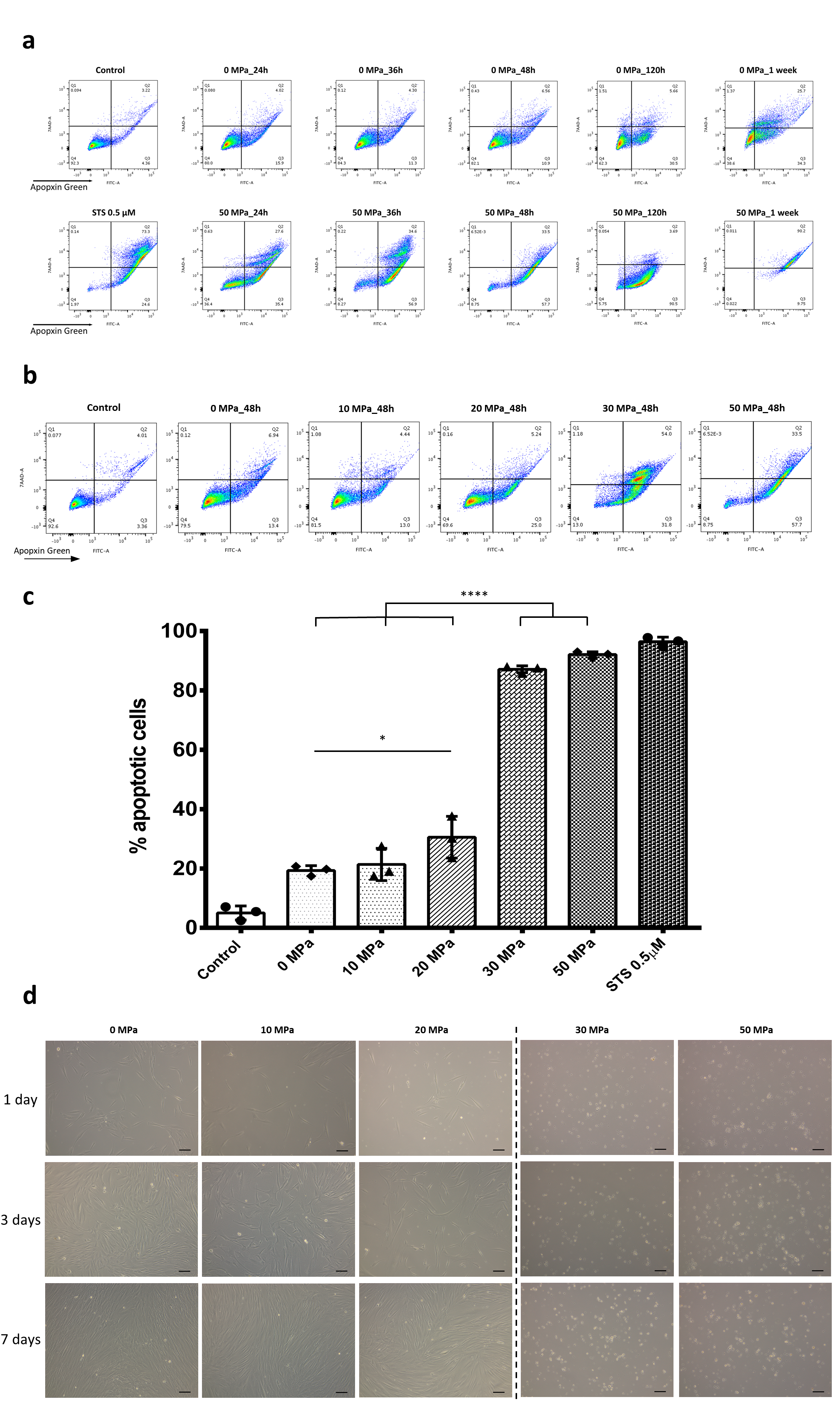

Supplement: Supplementary file 3 — Supplementary Figure S2. [file 41598_2020_74695_MOESM3_ESM.tif]
